# Supplementary material for: PRMT7 Inhibitor SGC8158 Enhances Doxorubicin-Induced DNA Damage and Its Cytotoxicity
Source: Int J Mol Sci. 2022 Oct 14;23(20):12323. doi: 10.3390/ijms232012323 (PMC9604017; doi:10.3390/ijms232012323)
Supplement: Supplementary file 1 [file ijms-23-12323-s001.zip › ijms-1957637-supplementary.pdf]

Supplementary data

PRMT7 inhibitor SGC8158 enhances doxorubicin-induced DNA damage and its cytotoxicity

Ahyeon Jeong[1], Yena Cho[1], Minkyeong Cho[1], Gyu Un Bae[1], Dae-Geun Song[2], Su-Nam Kim[2,3,*], and Yong Kee Kim[1,*]

[1] Muscle Physiome Research Center and Drug Information Research Institute, College of Pharmacy, Sookmyung Women's University, Seoul 04310, Republic of Korea

[2] Natural Products Research Institute, KIST Gangneung, Gangwon-do 25451, Republic of Korea

[3] Division of Bio-Medical Science and Technology, University of Science and Technology KIST School, Seoul 02792, Republic of Korea

* Correspondence: snkim@kist.re.kr (S.N.K.); Tel.: +82-33-650-3503, yksnbk@sookmyung.ac.kr (Y.K.K.); Tel.: +82-2-2077-7688

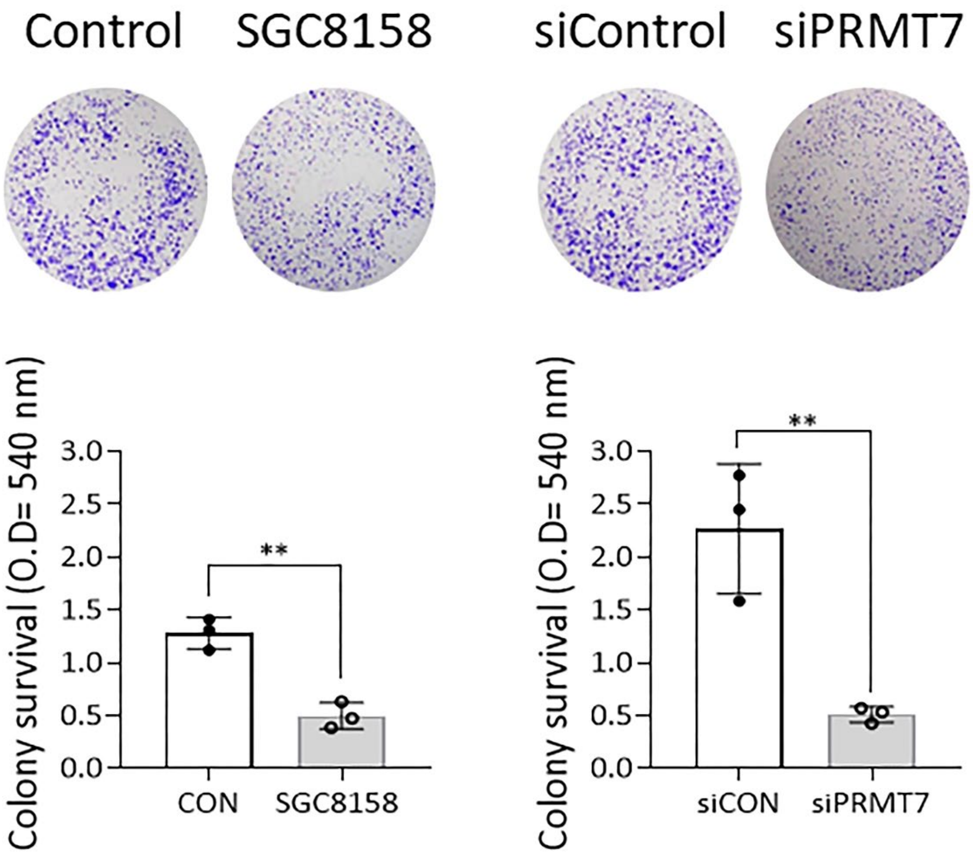

Figure S1. Suppression of PRMT7 attenuated colony formation of MCF7 cells. MCF7 cells were treated with 5 μM SGC8158 or transfected with PRMT7 siRNA as described. After ten days, the colonies were stained using crystal violet and the absorbance were measured at 540 nm. Data are presented as means ± SD (n = 3). **p < 0.01. SD: standard deviation

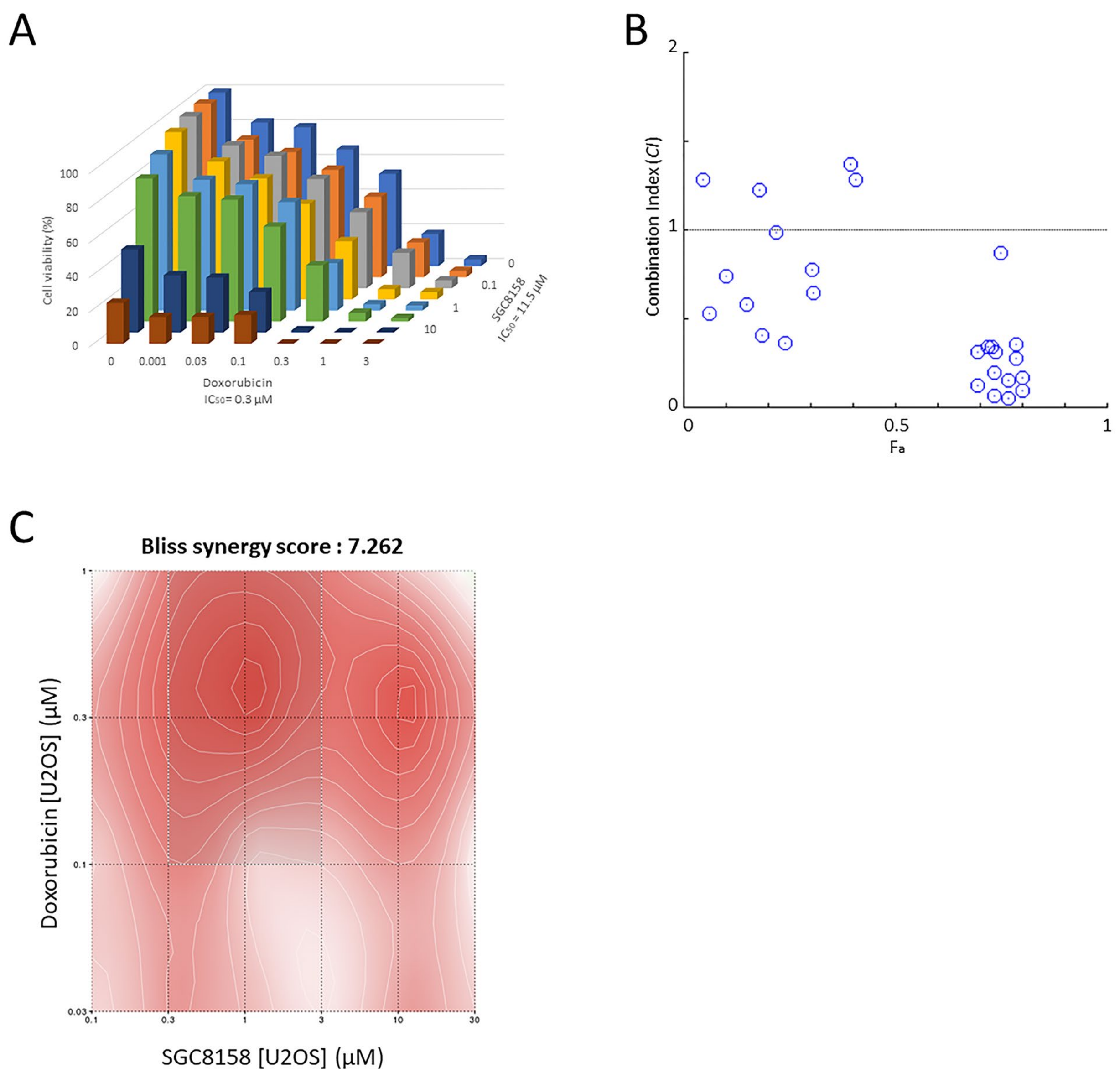

Figure S2. The synergistic effect of SGC8158 in combination with doxorubicin in U2OS cells. (A) In a combinatorial setting of SGC8158 and doxorubicin, the viabilities of U2OS cells were evaluated by MTT assay. (B) The CI values were calculated using CompuSyn software. (C) The Bliss synergy score was calculated using Bliss independent model. The gradation of the red regions indicates the intensity of synergism. MTT: 3-(4,5-dimethylthiazol-2-yl)-2,5-diphenyltetrazolium bromide; CI: combination index.
